# Mechanism Study of Traditional Medicine Using Proteomics Alone or Integrated with Other Systems Biology Technologies

**DOI:** 10.1155/2015/828159

**Published:** 2015-12-22

**Authors:** Xuan Liu, M. S. Kanthimathi, Klaus Heese

**Affiliations:** ^1^Shanghai Research Center for Modernization of TCM, Shanghai Institute of Materia Medica, Chinese Academy of Sciences, Shanghai 201203, China; ^2^Department of Molecular Medicine, Faculty of Medicine, University of Malaya, 50603 Kuala Lumpur, Malaysia; ^3^Graduate School of Biomedical Science & Engineering, Hanyang University, Seoul 133-791, Republic of Korea

The efficacy of traditional medicine such as traditional Chinese medicine (TCM) had been confirmed by many years of clinical use. However, the mechanisms of traditional medicine remained obscure. Since the development of systems biology in 2000, systems biology technologies have been popularly used in the mechanism study of traditional medicine. In this special issue, we particularly picked reviews and research papers related to mechanism study of TCM, TCM diagnosis, or TCM therapy methods using proteomics as well as other systems biology technologies. There are 10 papers collected in this special issue, in which 3 papers are reviews and 7 papers are original research papers.

The 3 reviews in the present issue introduce the advancements in applications of proteomics technologies in the study of TCM and the achievements in the mechanism study of TCM. Q. Ji et al. summarized the applications of proteomics technologies in TCM syndrome research as well as the mechanistic study of TCM treatments, including Chinese herbal medicine, Chinese herbal formula, and acupuncture. Furthermore, they introduced the combined analyses of proteomics with other “-omics” technologies in the study of TCM. Y. A. Sulistio and K. Heese discussed the utility of comparative proteomics for a better understanding of the mechanisms involved in TCM activities and its potential application as complementary therapy for the treatment of Alzheimer's disease (AD). Additionally, they reviewed the data from comparative proteomics studies of AD patients and established the relevance of the data with available AD hypotheses and potential TCM-based treatments, most notably regarding the ubiquitin proteasome system. Y. Yang et al. focused on discussing various results of proteomics studies of mechanisms of anticancer TCM, including terpenes, flavonoids, and glycosides. And they suggested future research strategies such as integrating translating mRNA analysis with proteomics in the study of the mechanisms of anticancer TCM.

Four of the research papers reported results of mechanism studies of TCM. J.-Y. Shiau et al. studied the mechanism of 2-*β*-D-glucopyranosyloxy-1-hydroxytrideca-5,7,9,11-tetrayne, an active component of* Bidens pilosa*, in human T-cell acute lymphocytic leukemia cells by using combined differential proteomics and bioinformatics approaches. M.-N. Shi et al. reported the protective effects of scutellarin, a flavone isolated from* Erigeron breviscapus* (Vant.) Hand.-Mazz, on human cardiac microvascular endothelial cells against hypoxia-reoxygenation injury. Possible target-related proteins of scutellarin were searched using proteomics analysis and a possible interaction network was predicted using bioinformatics analysis. C.-C. Guo et al. studied the antiosteoporotic effects and possible target-related proteins of Huangqi Sanxian decoction, a traditional Chinese formula composed of* Radix astragali*,* Epimedii folium*,* Cistanche herba*,* Radix notoginseng*,* Radix Salviae Miltiorrhiae*,* Corydalis rhizoma*,* Radix Angelicae sinensis*, and* Radix Clematidis*, in cultured rat osteoblasts. Z. Bo et al. investigated the molecular mechanism of fibroblast regulation and the treatment of recurrent oral ulcer by Shuizhongcao granule-containing serum. Shuizhongcao granule is a TCM formula composed of buffalo horn, urine sediment, callicarpa, and so forth, and it exhibited great efficacy in the clinical treatment of recurrent oral ulcer.

In the present issue, there are also 3 research papers reporting about the mechanisms of TCM used for the diagnosis of diseases or specialized TCM therapy methods such as moxibustion. In the TCM system, tongue diagnosis has been an important diagnostic method. X. Liu et al. used the GC/MS technology to determine the potential changes of metabolites and identify special metabolic biomarkers in the tongue coating of* H. pylori* infected chronic gastritis patients. W.-T. Dang et al. studied the expression of caspase-1 gene transcript variant mRNA in peripheral blood mononuclear cells of patients with primary gout in different TCM syndromes. X.-L. Shi et al. investigated the effects of moxibustion, a traditional Chinese practice that involves heated* Artemisia vulgaris* (mugwort) stimulation, on hormonal imbalance and ovarian granulosa cell apoptosis in a rat model of perimenopause.

In summary, this special issue provides a snapshot of the current status of mechanism studies of traditional medicine, especially TCM, using proteomics and other systems biology technologies. Authors of the present issue highlight both the achievements and challenges faced in the field of the mechanism study of traditional medicine. Hopefully, this publication will help readers to follow mechanism studies of traditional medicine and will contribute to improved applications of proteomics or other systems biology technologies in research of traditional medicine.

